# Correlation of Clinicohaematological Parameters in Paediatric Dengue: A Retrospective Study

**DOI:** 10.1155/2015/647162

**Published:** 2015-12-24

**Authors:** Ramakrishna Pai Jakribettu, Rekha Boloor, Andrew Thaliath, Sharanya Yesudasan George, Thomas George, Manoj Ponadka Rai, Umran Rafique Sheikh, Kadke Shreedhara Avabratha, Manjeshwar Shrinath Baliga

**Affiliations:** ^1^Department of Microbiology, Father Muller Medical College, Kankanady, Mangalore, Karnataka 575002, India; ^2^Father Muller Research Centre, Kankanady, Mangalore, Karnataka 575002, India; ^3^Department of Paediatrics, Father Muller Medical College, Kankanady, Mangalore, Karnataka 575002, India

## Abstract

Dengue is one of the arthropod-borne (arbo) viral diseases transmitted by female mosquito* Aedes aegypti*. Dengue fever has a wide spectrum of clinical presentation ranging from flu-like illness to severe complicated stage of dengue hemorrhagic fever leading to mortality. This was a retrospective study conducted in a tertiary care hospital in Coastal Karnataka, South India, to know the correlation between the clinical presentation and haematological parameters in the paediatric cases presented with dengue symptoms. A total of 163 paediatric cases who presented fever and dengue-like illness were included in the study. Of which, 69 were confirmed dengue patients. Critical analysis showed that there was a significant difference in the haematological parameters like total leucocyte count, percent differential leucocyte count, and platelets count, in the erythrocyte sedimentation rate (*P* < 0.05 to 0.0001). Additionally, when compared to nondengue patients, even the liver function and renal function parameters were significantly deranged (*P* < 0.05 to 0.0001). Stratification based on NS1, IgG, and IgM showed significant alterations in the haematological, hepatic, and renal parameters. With respect to the treatment a small percentage of patients, that is, 8% (4 patients), required platelet transfusion as their counts went below 20,000/*μ*L. Two patients succumbed to their illness while three required ICU stay.

## 1. Introduction

Recent global statistics indicate that the dengue virus, that causes the dengue fever, has spread wildly in the last four decades to more than 100 countries in the tropical and subtropical areas [1–3]. It is arthropod-borne viral disease with high morbidity and mortality and is today considered to be of importance to public health [2, 3]. Epidemiologically, it is estimated that dengue infects more than 50 million people each year and around two and a half billion people are at risk of infection [4]. The female* Aedes aegypti* and* Aedes albopictus* mosquitoes transmit the virus during the blood meal. Dengue virus is a single stranded positive sense RNA virus belonging to Flaviviridae family and reports indicate that there are 4 serotypes (1, 2, 3, and 4).

Dengue is an acute infection, in which majority of cases may be asymptomatic or may have a wide spectrum of clinical symptoms, ranging from nonspecific flu-like symptoms (dengue fever) with biphasic fever, skin rashes, mild-to-moderate myalgia, retro-orbital pain, and arthralgia [2, 3]. The severe form will have disseminated intravascular coagulopathy (dengue haemorrhagic fever) which may progress to hypovolemic shock (dengue shock syndrome (DSS)) [5–7]. Innumerable reports have strongly shown that the dengue hemorrhagic fever (DHF), characterized by thrombocytopenia due to hemorrhagic manifestations, increased vascular permeability leading to depletion of the intravascular volume and shock that cumulatively leads to irreversible multiorgan failure (liver first, then kidney, heart, and brain) and results in death [6, 8]. However this classification was difficult to apply in all clinical settings. The new WHO classification of children with dengue fever proposes (i) dengue fever and (ii) severe dengue. The dengue fever group is further categorised as with or without warning signs [9].

Laboratory diagnosis of dengue infection is similar to any other viral infection; it includes viral isolation, detection of viral nucleic acid, antigens, or antibodies. In the initial stage of infection, that is, first 4-5 days, dengue virus can be isolated from the plasma, serum, and circulating blood cells. The immunological response to the viral antigen of dengue depends on the immune status of the infected person. In primary dengue, that is, when dengue has infected a person who has never been infected or immunised against any flavivirus, then there will be a primary antibody response with production of specific IgM antibody. The detectable IgM will be produced by as early as 3–5 days of illness, reaching peak by 10 days and decline to undetectable levels by 2-3 months. The anti-dengue IgG antibody will be appearing in the first week of illness and later remain detectable for several months and even for life [10]. In secondary infection (i.e., when a host has already had a previous episode of infection of dengue virus or is vaccinated against any flavivirus), there will be rapid rise in antibody titres which react against many flaviviruses. In such cases the anti-dengue IgG antibody is detected in the acute phase of infection and this persists for a long duration of time. The IgM antibody in secondary dengue infection is significantly lower and undetectable than primary dengue infection. This necessitates the identification of both anti-dengue IgM and anti-dengue IgG antibodies to confirm secondary dengue infection [10]. A viral nonstructural protein, NS1, is released by infected cells into circulation and can be detected using monoclonal or polyclonal antibodies. The detection of NS1 is the basis of commercial tests, including rapid tests. These tests offer reliable point of care diagnosis of acute dengue infection [11].

In Asia, the effects of dengue infection and possibility of developing severe disease are more in children below the age of 15 than in the adults [12, 13]. Dengue fever is known to affect haematological parameters and accordingly a simple clinical and haematological monitoring of the afflicted patients helps to reduce the morbidity and mortality. To validate this hypothesis, the present study was taken up in people attending a tertiary care centre at Coastal Karnataka, India.

## 2. Materials and Methods

This was a retrospective study and was conducted in the Department of Clinical Microbiology and Paediatrics, Father Muller Medical College Hospital, Mangalore, during January 2013 to March 2014. The study was approved by the institutional ethics committee. All patients below the age of 18 years, who got admitted with the history of fever and suspicion of dengue, were included in the study. The exclusion criteria included children with confirmed reports of malaria, cancer, tuberculosis, HIV, and bacterial and parasitic illness and those who were on any medication (antibiotic, antipyretics, anti-inflammatory) for the past two months. The serological assays for dengue were performed using standard kit (J. Mitra & Co. Pvt. Ltd., New Delhi). Children with positive result for NS1Ag or IgM or IgG antibodies against dengue virus were considered dengue-positive group, while those that were not positive for the three assays were considered dengue negative. All the clinical, haematological, and treatment details during the study time period were considered.

The data from individual patients satisfying the inclusion and exclusion criteria were noted down from individual files and entered into the Microsoft Excel. The demographic details were categorised into frequency, while the hematological and biochemical data were calculated to obtain mean ± standard deviation (SD). All these details are represented in the represented in each of the tables. For overall comparison, results were compared as dengue negative and dengue positive and subjected to the Student *t*-test. Additionally a, subclassification of the dengue positive cases was also done based on the results of the NS1Ag, IgM, and IgG. These data were subjected to one-way ANOVA and post-ANOVA (Bonferroni-Holm) test to compare for the fine changes. A *P* value of 0.05 was considered significant.

## 3. Results

**Figure 1:**
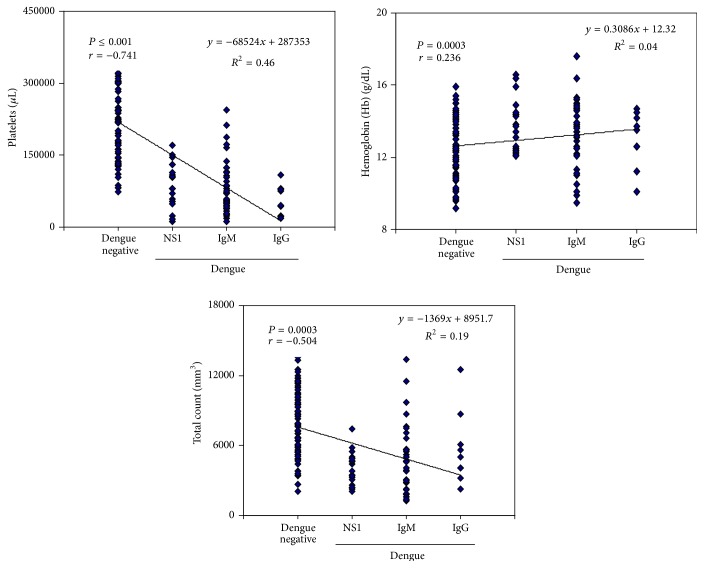
Association of platelets, haemoglobin, and total count with dengue negative, NS1, IgG, and IgM.

A total of 163 paediatric cases who presented with fever and dengue-like illness were included in the study. Of these, 69 were confirmed dengue cases and remaining 94 were considered as dengue negative and appropriate control. Among the positive dengue cases, 69.56 (48/69) were male and 30.43 (21/69) were female children (Table 1). The patients presented with various concomitant clinical features the most prominent being fever, vomiting, myalgia, rashes, haematuria, epistaxis, and subconjunctival haemorrhage (details enlisted in Table 1). With respect to the haematological parameters, a significant difference in the total leucocyte count, percent differential leucocyte count, and platelets count and in the erythrocyte sedimentation rate was observed (Tables 2 and 3). Correlation of the platelets, haemoglobin, and total count with dengue negative, NS1, IgG, and IgM showed an association and is represented in Figure 1. Significant differences were also seen in the liver function test and in renal parameters and are expressed in Tables 4 and 5. All patients were treated with intravenous fluid and antipyretics (Table 6). Antibiotics were prescribed to 13 patients (26%) to prevent secondary bacterial infection during the hospital stay (Table 6). A small percentage of patients, that is, 8% (4 patients), required platelet transfusion as their counts went below 20,000/*μ*L.

Three patients (4%) required ICU stay as their condition when brought to the hospital was critical. Of these two children, both girls of age 9 succumbed to the disease. At the time of admission these children presented with acute atypical symptoms of fever with vomiting and abdominal pain of 3-day duration. One girl presented with abdominal pain whose ultrasonography of abdomen showed hepatosplenomegaly, moderate ascites, and pleural effusion. Haematological investigation showed anaemia and thrombocytopenia due to bone marrow depression. During the course of hospitalization, she developed respiratory distress and acute renal failure and this leads to her death. Second girl also admitted with vomiting and pain abdomen was diagnosed to have severe thrombocytopenia with platelet count of 19,000/cmm and later had pulmonary haemorrhage and was the causative factor for the death. Both of the patients were transfused with platelet concentrate, 3 and 2 pints, respectively, but in spite of best of the efforts to maintain hemostasis, the children succumbed to death.

## 4. Discussion

The main presenting illness in our patients was fever (100%), myalgia (100%), headache (52.16%), and vomiting (42.03%). These observations are contradictory to the observations of previous studies where 85% [14] and 33% [15] of patients presented with fever. On the contrary, when compared to previous studies [14, 15] myalgia was seen in only 42% of the patients as against 81% and 84.5%. Additionally, when compared to earlier studies the incidence of haematuria, subconjunctival haemorrhage, epistaxis, pain in abdomen, GI bleeding, and petechiae was also less [16–18] indicating that the difference in the clinical manifestation is possibly due to difference in the strain of the virus and its virulence factor.

In people afflicted with dengue bone marrow suppression, causes haematological features like leucopenia and neutropenia. In our study it was observed that the dengue patients had a mean total leucocyte count of 4,984 ± 3,082 as compared to 7,926 ± 2,760 of dengue negative cases. Neutrophils counts were 49 ± 19, less compared to 63 ± 15 of dengue negative cases [14, 19]. Leucopenia was present in 26% in Ratageri study and in 66% in a study from Eluru, Andhra [20]. The haemoglobin count was more in dengue patients; this may be attributed to hemoconcentration due to increased intravascular permeability. Hematocrit though more specific not initially done in most of the cases.

 In secondary dengue there are higher chances of development of DHS/DSS and paediatric patients are at higher risk than adults [1]. The increased incidence of DHS in children may be due to greater baseline microvascular permeability compared to adults. We had similar observation like Banerjee et al. 2008 [14], having no case of DHS. This may be due to the difference in the population under study. The platelets counts were significantly low compared to dengue negative cases as shown in Table 2, compared to similar observation of earlier investigators [14, 16, 17, 19]. Similar observations were made by others in various parts of India [18, 20, 21]. Though clinical jaundice is rare pain abdomen due to serositis is known. Liver enzymes are significantly altered in dengue positive cases compared to controls and earlier reports do indicate that the transiently elevated transaminase levels returned to normal after 4 weeks [21].

Dengue fever is a self-limiting arboviral infection transmitted by vectors* Aedes aegypti *and* Aedes albopictus*. Thorough physical examination, monitoring of haematological parameters is sufficient to prevent high mortality and morbidity in dengue fever especially in paediatric patients. According to the Indian Ministry of Health and Family Welfare, 2013 was the worst year with total of 75,454 dengue cases reported all over India and death troll reaching 167. In 2015 till 29 May, 3816 cases with 9 deaths have been reported [22]. However, in this study there was no significant mortality in the present study, probably due to increase in awareness, early diagnosis, and proper management and, as mentioned earlier, population type. Presently there is no specific anti dengue treatment available. Hence, symptomatic treatment especially for electrolyte imbalance and fluid loss is the only treatment recommended [2]. Like any vector borne disease, the best method to prevent dengue is by control of vectors* Aedes aegypti* and* Aedes albopictus*.

## Figures and Tables

**Table 1:** Demographic and clinical symptoms of the children with dengue [percent (actual numbers)].

Demographic and patient details	Dengue negative	Dengue positive
Males	61.7% (58/94)	69.56 (48/69)
Females	38.3% (36/94)	30.43 (21/69)
Average age (in years)	11.12 ± 4.75	13.61 ± 4.9
Average hospital stay (in days)	3.14 ± 1.5	5.12 ± 1.9
ICU stay	5.31 (5/94)	6.34 (3/69)
Death	Nil	2.9 (2/69)
Discharge	100 (94/94)	97.1 (67/69)

Signs and symptoms on presentation	Dengue negative	Dengue positive

Fever	100 (94/94)	100 (69/69)
Myalgia	64.89 (61/94)	100 (69/69)
Headache	36.17 (34/94)	52.17 (36/69)
Vomiting	45.74 (43/94)	42.03 (29/69)
Rashes	7.44 (7/94)	18.84 (13/69)
Cough	52.12 (49/94)	13.04 (9/69)
Pain abdomen	55.31 (52/94)	11.59 (8/69)
Loose stools	64.48 (61/94)	8.69 (6/69)
Haematuria	0	2.90 (2/69)
Epistaxis	0	1.45 (1/69)
Bleeding from gums	0	1.45 (1/69)
Hepatomegaly	4.26 (4/94)	4.35 (3/69)
Splenomegaly	6.38 (6/94)	2.90 (2/69)

**Table 2:** Alterations in haematological parameters in dengue-positive and dengue-negative paediatric cases.

	Dengue negative	Dengue positive	*P* value
	(*n* = 94)	(*n* = 69)	
HB	12.52 ± 1.48 (9.2–15.9)	13.39 ± 1.68 (9.5–17.6)	0.0006 HS

TC	8448.2 ± 5479.2 (2100–13600)	4583.74 ± 2548 (1300–13400)	<0.0001 HS

Neutrophils	63.26 ± 14.82 (36–92)	50.36 ± 18.17 (3–87)	<0.0001 HS

Lymphocytes	30.13 ± 14.022 (5–76)	40.98 ± 17.75 (3–78)	<0.0001 HS

Eosinophils	4.42 ± 5.0891 (1–26)	1.95 ± 1.68 (0–8)	<0.0001 HS

Monocytes	2.41 ± 1.66 (0–11)	4.55 ± 2.77 (0–12)	<0.0001 HS

Platelets	225175.02 ± 86163.35 (73000–388000)	81159.42 ± 50131.61 (12000–245000)	<0.0001 HS

**Table 3:** Alterations in the haematological parameters by comparing with different stages of dengue.

	Dengue negative (*n* = 94)	NS1 (*n* = 21)	IgM (*n* = 39)	IgG (*n* = 09)	*P* value
HB	12.52 ± 1.48 (9.2–15.9)	13.81 ± 1.47^$^ (12.1–16.6)	13.24 ± 1.81 (9.5–17.6)	13.01 ± 1.46 (10.1–14.7)	0.003 HS

TC	7925.6 ± 2760.3 (2100–13600)	4023.80 ± 1323.4^*∗*^ (2100–7400)	4621.00 ± 2844.7^*∗*^ (1300–13400)	5733.33 ± 2959.3 (2300–12500)	0.0001

Neutrophils	63.26 ± 14.82 (36–92)	50.19 ± 16.27^%^ (23–83)	50.38 ± 17.91^*∗*^ (3–87)	50.66 ± 22.87 (22–82)	0.0001 HS

Lymphocytes	30.13 ± 14.022 (5–76)	41.61 ± 16.82^#^ (5–75)	41.1 ± 17.62^&^ (3–78)	39.0 ± 20.09 (14–66)	0.001 HS

Eosinophils	4.42 ± 5.0891 (1–26)	1.61 ± 1.09^@^ (0–4)	2.07 ± 1.76^#^ (1–8)	2.22 ± 2.25 (2–8)	0.003 HS

Monocytes	2.41 ± 1.66 (0–11)	5.71 ± 3.58^*∗*^ (0–12)	3.61 ± 1.90^#^ (1–8)	5.88 ± 2.07^*∗*^ (2–8)	0.0001 HS

Platelets	225175.0 ± 86163.3 (73000–388000)	88571.4 ± 42819^*∗*^ (12000–170000)	83154.0 ± 55327^*∗*^ (12000–245000)	55222 ± 30106^*∗*^ (19000–109000).	0.0001 HS

Statistical comparison of the various dengue groups with the dengue-negative ANOVA with Bonferroni's multiple comparison  [^*∗*^ *P* < 0.0001; ^$^ *P* ≤ 0.004;  ^%^ *P* < 0.008; ^&^ *P* < 0.002; ^#^ *P* < 0.02; ^@^ *P* < 0.03]. Values in bracket below each value is the lower and upper bound.

**Table 4:** Alterations in the biochemical parameters of liver and kidney functioning in the dengue-positive and dengue-negative cases in children.

	Dengue negative	Dengue positive	*P* value
T. bilirubin	0.62 ± 0.40 [*n* = 43] (0.2–2.1)	0.74 ± 0.89 [*n* = 39] (0.17–3.6)	0.42

Conjugated bilirubin	0.21 ± 0.11 [*n* = 34] (0.1–1.6)	0.50 ± 0.79 [*n* = 30] (0.1–2.8)	0.052

Unconjugated bilirubin	0.36 ± 0.23 [*n* = 34] (0.1–1.1)	0.32 ± 0.29 [*n* = 29] (0.1–1.2)	0.53

SGOT	28.36 ± 11.97 [*n* = 46] (13–64)	134.43 ± 145.8 [*n* = 39] (14–696)	<0.0001 HS

SGPT	5.12 ± 20.08 [*n* = 48] (10–115)	88.64 ± 98.49 [*n* = 40] (10–4728)	<0.0001 HS

Urea	20.74 ± 8.29 [*n* = 43] (10–38)	20.18 ± 7.54 [*n* = 38] (1–39)	0.75

Creatinine	0.75 ± 0.25 [*n* = 45] (0.5–1.4)	0.73 ± 0.22 [*n* = 46] (0.32–1.3)	0.79

**Table 5:** Alterations in biochemical parameters by comparing with different stages of dengue.

	Dengue negative	NS1	IgM	IgG	*P* value
T. bilirubin	0.62 ± 0.40 [*n* = 43] (0.2–2.1)	0.30 ± 0.11 [*n* = 11] (0.17–0.58)	0.87 ± 0.93 [*n* = 23] (0.2–3.4)	1.11 ± 1.25 [*n* = 5] (0.39–3.6)	0.05 HS

Conjugated bilirubin	0.21 ± 0.11 [*n* = 34] (0.1–1.6)	0.13 ± 0.04 [*n* = 7] (0.1–0.2)	0.55 ± 0.83 [*n* = 19] (0.1–2.8)	0.82 ± 1.03 [*n* = 4] (0.16–2.6)	0.042 HS

Unconjugated bilirubin	0.36 ± 0.23 [*n* = 34] (0.1–1.1)	0.18 ± 0.10 [*n* = 6] (0.1–0.4)	0.35 ± 0.31 [*n* = 19] (0.1–1.2)	0.37 ± 0.36 [*n* = 4] (0.1–1.0)	0.492

SGOT	28.36 ± 11.97 [*n* = 46] (13–64)	123.72 ± 126.2^∧^ [*n* = 11] (23–442)	145.0 ± 160.66^*∗*^ [*n* = 23] (14–696)	91.44 ± 108.62 [*n* = 5] (44–305)	0.0001 HS

SGPT	25.12 ± 20.08 [*n* = 48] (10–115)	93.12 ± 91.51 [*n* = 6] (10–360)	280.1 ± 893.02 [*n* = 28] (13–4728)	66.33 ± 61.03 [*n* = 6] (14–192)	0.187

Urea	20.74 ± 8.29 [*n* = 43] (10–38)	22.45 ± 8.52 [*n* = 11] (11–39)	18.5 ± 6.85 [*n* = 22] (1–30)	19.0 ± 10.62 [*n* = 5] (10–36)	0.575

Creatinine	0.75 ± 0.25 [*n* = 45] (0.5–1.4)	0.72 ± 0.24 [*n* = 12] (0.32–1.3)	0.7 ± 0.19 [*n* = 28] (0.5–1.2)	0.92 ± 0.26 [*n* = 6] (0.54–1.3)	0.22

Statistical comparison of the various dengue groups with the dengue-negative ANOVA with Bonferroni's multiple comparison  [^*∗*^ *P* < 0.0001; ^∧^ *P* < 0.04]. Values in bracket below each value is the lower and upper bound.

**Table 6:** Treatment administered to the children with dengue.

Treatment	Percent (actual numbers)
IV fluids	100 (69/69)
Antipyretics	100 (69/69)
Antibiotics	27.53 (19/69)
Vitamin supplementation	18.84 (13/69)
Antiemetics	18.84 (13/69)
Antacids	15.94 (11/69)
Platelet transfusion	11.59 (8/69)

## References

[1] Guzmán M. G., Kouri G. (2002). Dengue: an update. *The Lancet Infectious Diseases*.

[2] Halstead S. B., Schlesinger R. W. (1980). Immunological parameters of togavirus disease syndromes. *The Togaviruses*.

[3] Halstead S. B. (1988). Pathogenesis of dengue: challenges to molecular biology. *Science*.

[4] Guha-Sapir D., Schimmer B. (2005). Dengue fever: new paradigms for a changing epidemiology. *Emerging Themes in Epidemiology*.

[5] Harris E., Videa E., Pérez L. (2000). Clinical, epidemiologic, and virologic features of dengue in the 1998 epidemic in Nicaragua. *The American Journal of Tropical Medicine and Hygiene*.

[6] Rajapakse S. (2011). Dengue shock. *Journal of Emergencies, Trauma and Shock*.

[7] World Health Organization (1975). *Technical Guides for Diagnosis, Treatment, Surveillance, Prevention and Control of Dengue Hemorrhagic Fever*.

[8] Sam S.-S., Omar S. F. S., Teoh B.-T., Abd-Jamil J., AbuBakar S. (2013). Review of Dengue hemorrhagic fever fatal cases seen among adults: a retrospective study. *PLoS Neglected Tropical Diseases*.

[9] World Health Organization (2012). *Handbook for Clinical Management of Dengue*.

[10] World Health Organization (1997). *Dengue Haemorrhagic Fever: Diagnosis, Treatment, Prevention and Control*.

[11] Halstead B. S. (2011). Denguefever and dengue hemorrhagic fever. *Nelson Textbook of Pediatrics*.

[12] Kittigul L., Pitakarnjanakul P., Sujirarat D., Siripanichgon K. (2007). The differences of clinical manifestations and laboratory findings in children and adults with dengue virus infection. *Journal of Clinical Virology*.

[13] Hung N. T., Lei H.-Y., Lan N. T. (2004). Dengue hemorrhagic fever in infants: a study of clinical and cytokine profiles. *Journal of Infectious Diseases*.

[14] Banerjee M., Chatterjee T., Choudhary G. S., Srinivas V., Kataria V. K. (2008). Dengue: a clinicohaematological profile. *Medical Journal Armed Forces India*.

[15] Pervin M., Tabassum S., Ali M., Kazi M. (2004). *Clinical and Laboratory Observations Associated with the 2000 Dengue Outbreak in Dhaka, Bangladesh*.

[16] Singh N. P., Jhamb R., Agarwal S. K. (2005). The 2003 outbreak of dengue fever in Delhi, India. *Southeast Asian Journal of Tropical Medicine and Public Health*.

[17] Hussin N., Jaafar J., Naing N. N., Mat H. A., Muhamad A. H., Mamat M. N. (2005). A review of dengue fever incidence in Kota Bharu, Kelantan, Malaysia during the years 1998–2003. *Southeast Asian Journal of Tropical Medicine and Public Health*.

[18] Ratageri V. H., Shepur T. A., Wari P. K., Chavan S. C., Mujahid I. B., Yergolkar P. N. (2005). Clinical profile and outcome of dengue fever cases. *Indian Journal of Pediatrics*.

[19] Ahmed S., Arif F., Yahya Y. (2008). Dengue fever outbreak in Karachi 2006—a study of profile and outcome of children under 15 years of age. *Journal of the Pakistan Medical Association*.

[20] Prathyusha C. V., Rao M. S., Sudarsini P., Rao K. M. (2013). Clinico-haematological profile and outcome of dengue fever in children. *International Journal of Current Microbiology and Applied Sciences*.

[21] Sharma P., Kumar C. M., Patwari A. K. (2014). Clinical profile of early diagnosed dengue fever in hospitalized children in south Delhi. *The Indian Journal of Pediatrics*.

[22] http://nvbdcp.gov.in/den-cd.html.

